# Bone Marrow Findings in Patients With Renal Disease: Experience of a Single Tertiary Care Center in Bihar

**DOI:** 10.7759/cureus.83900

**Published:** 2025-05-11

**Authors:** Rawi Agrawal, Satish Kumar, Kaushal Kumar, Vijayanand Choudhary

**Affiliations:** 1 Department of Hematology, Indira Gandhi Institute of Medical Sciences, Patna, IND

**Keywords:** anemia, bone marrow examination, chronic kidney disease (ckd), dyspoiesis, hematopoiesis, plasma cell disorders

## Abstract

Background

This study aimed to characterize bone marrow findings in patients with chronic kidney disease (CKD) in Bihar, India, and to identify correlations between these findings and patients’ clinical presentations and hematological parameters. Specifically, this study aimed to (1) document the prevalence and patterns of bone marrow abnormalities in CKD patients, (2) analyze associations between bone marrow findings and CKD stages, and (3) investigate relationships between bone marrow changes and hematological parameters, including anemia severity and morphology.

Methodology

A retrospective, observational study was conducted by analyzing bone marrow findings of 220 CKD cases admitted at a tertiary care hospital in Bihar, India. Patients’ demographic details, clinical features, and laboratory data, including complete blood count, peripheral blood smear, renal function tests, and serum protein electrophoresis, were documented. Standard techniques were utilized to perform bone marrow aspiration and biopsy, which were, subsequently, analyzed by two independent hematopathologists. The data were analyzed for correlations between CKD stages, anemia severity, bone marrow findings, and other clinical parameters.

Results

The study population was predominantly middle-aged males (63.2%) with a high burden of hypertension (90%) and diabetes (46.4%). All patients presented with anemia, with moderate-grade anemia being the most common (45.9%), and the normocytic, normochromic being the most prevalent subtype (48.6%). Bone marrow examination revealed normal trilineage hematopoiesis in 44.1% of cases. Other notable findings included increased plasma cells (23.2%), erythroid hyperplasia (17.7%), and features of dyspoiesis (8.2%). More advanced stages of CKD were associated with greater plasma cell disorder and dyspoietic feature dominance along with erythroid hyperplasia and lower rates of normal hematopoiesis. Dialysis-dependent patients showed higher rates of bone marrow abnormalities compared to non-dialysis CKD patients.

Conclusions

This study establishes the reference norms for bone marrow changes in CKD patients in Bihar and demonstrates an elaborate spectrum of hematological changes beyond mere renal anemia. The increased prevalence of plasma cell disorders and dyspoietic changes, especially in advanced CKD, underscores the importance of bone marrow examination in patients with undiagnosed cytopenias and suspected paraproteinemias.

## Introduction

Chronic kidney disease (CKD) poses a formidable public health problem worldwide, especially in developing nations. In India, the prevalence of CKD has been estimated to be around 17.2%, which is considerably higher than that of the United States (13.9%), China (10.8%), Japan (12.9%), and Brazil (8.9%). In India, prevalence shows considerable intra-country regional differences, with the southern zone reporting a prevalence of 14.78%, rural populations 15.34%, and urban areas 10.65% [1].

Among the various pathological manifestations of CKD, hematological complications, specifically anemia, are among the most clinically significant. In addition to reduced erythropoietin production, the pathophysiological mechanisms of anemia in CKD include uremic repression of erythropoiesis, increased red blood cell destruction, iron and folate deficiency, blood loss from dialysis or uremia, and hypoplastic bone marrow. These conditions cause severe deterioration linked to the steady progression of renal disease, which significantly lowers clinical results and patients’ quality of life [2].

Although the peripheral blood alterations within CKD patients have been rather well explored [3], there remains a scarcity of systematic research examining the bone marrow morphology and functionality within this population, especially in resource-limited settings such as Bihar, India. Bone marrow examination can provide vital information regarding the pathologic processes involved in hematopoiesis and can demonstrate pathologic processes that may not be evident with routine peripheral blood examination. Evaluating changes in bone marrow of CKD patients becomes necessary in distinguishing renal anemia from other coexisting hematologic disorders of the patient for determining proper management measures while diagnosing factors that can contribute to the acceleration of kidney disease progression [4].

Several bone marrow changes have been noted in patients with CKD that include some degree of erythroid hypoplasia or hyperplasia, dyserythropoiesis, myelofibrosis, increased plasma cell infiltration, and, in some cases, the presence of existing hematologic malignancies. The prevalence and spectrum of these findings are heterogeneous across various studies, which is most likely due to differences in the population of patients, etiology of CKD, stage distribution, treatment modalities, and some regional factors. Other existing comorbid conditions such as diabetes mellitus and hypertension, which are widely common in the CKD population, may also have an impact on the bone marrow response to uremia [5].

The high population density of Bihar makes it one of India’s most economically neglected states. These states are known to have complex and problematic healthcare pathways, such as a dearth of epidemiological data on CKD and associated hematologic consequences. The management of CKD in this area may be impacted by variables such access to healthcare, disease concentration, food, and environment. Based on the available literature, no thorough documentation of bone marrow findings of CKD patients in this area has been performed [6].

The first assessment of bone marrow features of CKD patients was conducted at a tertiary care hospital in Bihar, addressing the significant void in the regional hematology literature. Understanding the range of bone marrow changes and the clinical and laboratory relationships between them can help clarify how kidney disease and hematopoiesis interact. These modifications will enhance the results for CKD patients in this underserved area, allow for better techniques for controlling anemia, and facilitate better detection of associated diseases.

## Materials and methods

Study design and setting

The retrospective, observational study was conducted at the Indira Gandhi Institute of Medical Sciences, a tertiary care center located in Patna, Bihar. The study was exempted from the Institutional Ethics Committee review (reference number: 1371/IEC/IGIMS/2024). The study duration was from March 2024 to February 2025.

Study population

The study sample comprised 220 patients with clinically diagnosed CKD who underwent bone marrow examination for one or more clinical indications and were referred from outside the institute.

Inclusion and exclusion criteria

Regardless of gender, patients above 10 years of age who were documented to suffer from an advanced stage of CKD and triaged to have kidney damage, evidenced by glomerular filtration rate (GFR) declining to below 59 mL/minute/1.73 m² for a period exceeding three months, were included. Patients with a skin infection or recent radiation therapy to the sampling site’s skin and patients with bleeding conditions such as osteoporosis, osteomyelitis, or osteogenesis imperfecta were excluded due to contraindications associated with a bone marrow biopsy. All patients who refused to provide informed consent, as well as patients classified as having severe thrombocytopenia (platelet count <0.40 lac/mm^3^), were also excluded. Individuals with incomplete clinical or laboratory data, those with main hematological diseases diagnosed before the diagnosis of CKD, and those who had undergone blood transfusions within three weeks before the bone marrow examination were other exclusions.

Methodology

**Figure 1:**
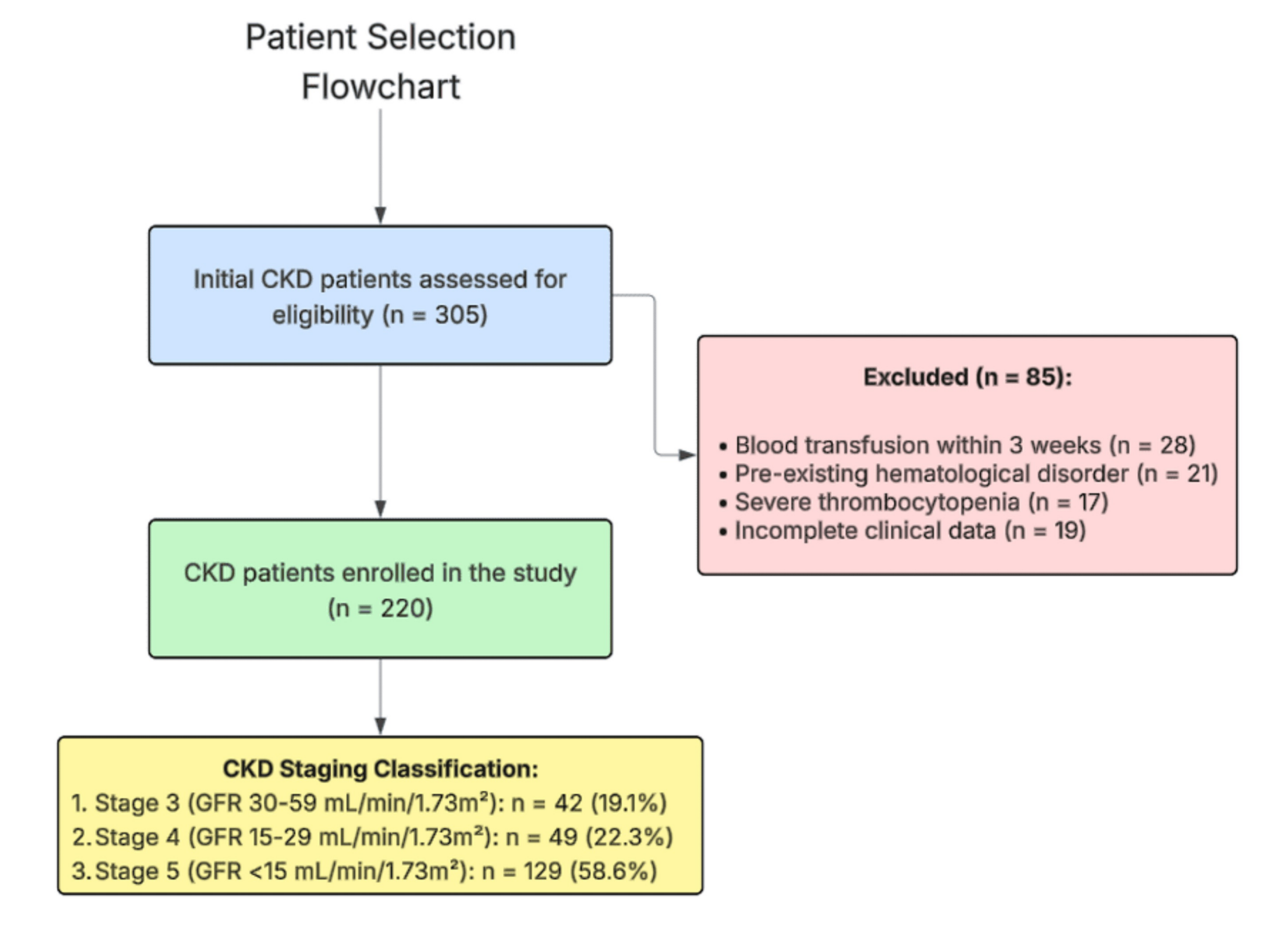
Flowchart depicting the systematic process of patient selection for the study. CKD: chronic kidney disease; GFR: glomerular filtration rate

CKD staging was performed according to the 2012 Kidney Disease: Improving Global Outcomes Clinical Practice Guidelines, with patients classified based on their estimated glomerular filtration rate (eGFR) calculated using the CKD-EPI equation. Stage 3 CKD was defined as an eGFR of 30-59 mL/minute/1.73m², Stage 4 as an eGFR of 15-29 mL/minute/1.73m², and Stage 5 as an eGFR of <15 mL/minute/1.73m². Patient selection followed a systematic approach as detailed in Figure 1, with 305 initially assessed patients narrowed to the final study population of 220 after applying the exclusion criteria.

For statistical analysis, descriptive statistics included frequencies and proportions for categorical variables. The chi-square goodness-of-fit test was used to compare observed distributions against theoretically expected equal distributions. The chi-square test for independence was employed when analyzing associations between CKD stages and bone marrow findings, with Bonferroni corrections applied for multiple comparisons. Two-tailed binomial confidence intervals were calculated using the Clopper-Pearson method with 95% confidence levels. Statistical significance was set at p-values <0.05 for all analyses. Post-hoc power analysis confirmed >80% power to detect medium effect sizes (w = 0.3) in the primary chi-square analyses.

A comprehensive data collection approach was employed to establish connections between renal disease progression and hematological manifestations, with a particular focus on bone marrow alterations. Detailed demographic and clinical information was recorded, including systemic symptoms (fever, weight loss, weakness/lethargy/fatigue), comorbidities (hypertension, diabetes, ischemic heart disease), physical examination findings (hepatomegaly, splenomegaly, lymphadenopathy), and bleeding manifestations (petechiae/purpura). Laboratory parameters assessed included complete blood count for grading anemia severity, platelet count assessment, and white blood cell evaluation. Peripheral blood smear analysis categorized anemia morphology as normocytic normochromic, microcytic hypochromic, macrocytic, or dimorphic. Serum protein electrophoresis was performed to detect paraproteinemias, with results classified as normal, showing distortion, or presenting M-spike patterns.

**Figure 2:**
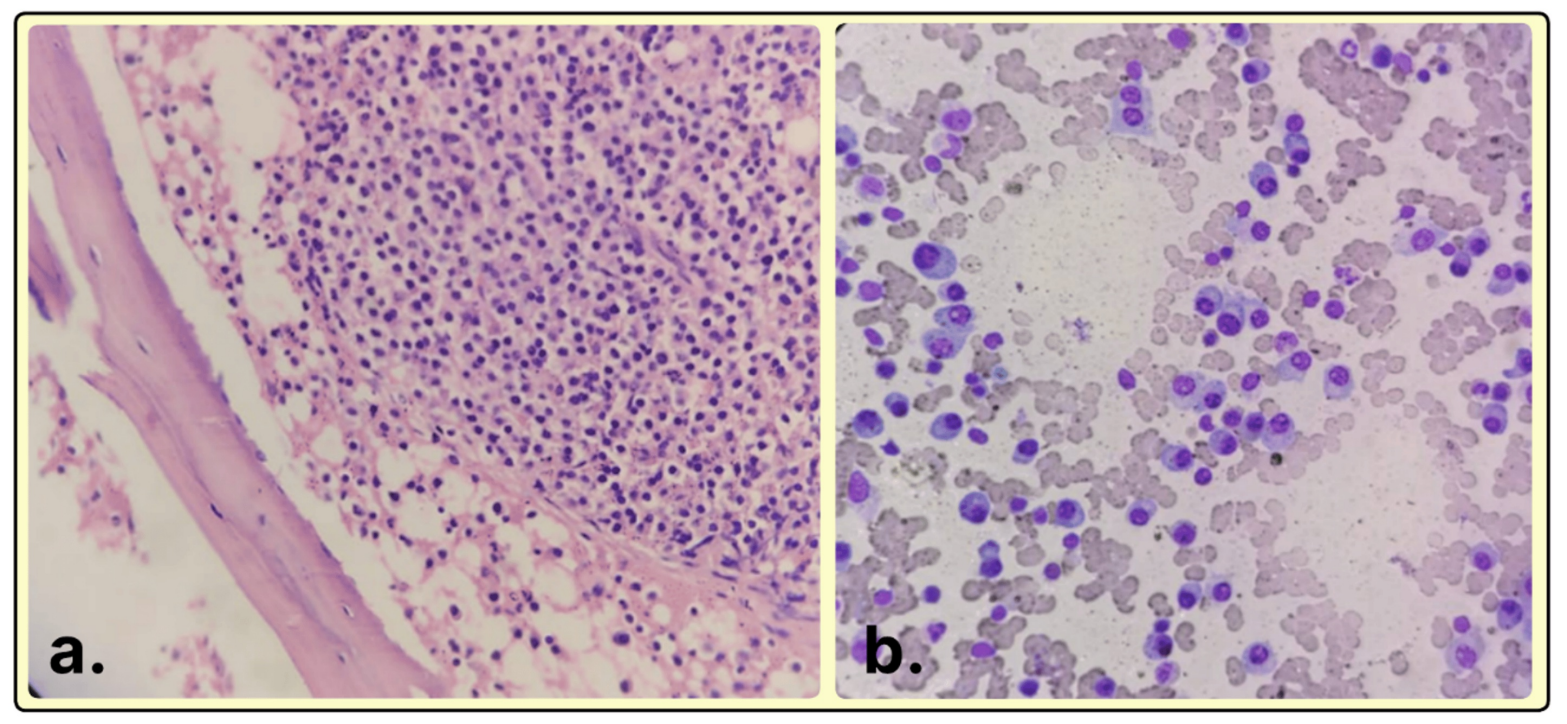
(a) Bone marrow biopsy showing plasma cells in sheets (hematoxylin and eosin, ×100). (b) Bone marrow aspirate showing the presence of >60% plasma cells (Leishman, ×400).

**Figure 3:**
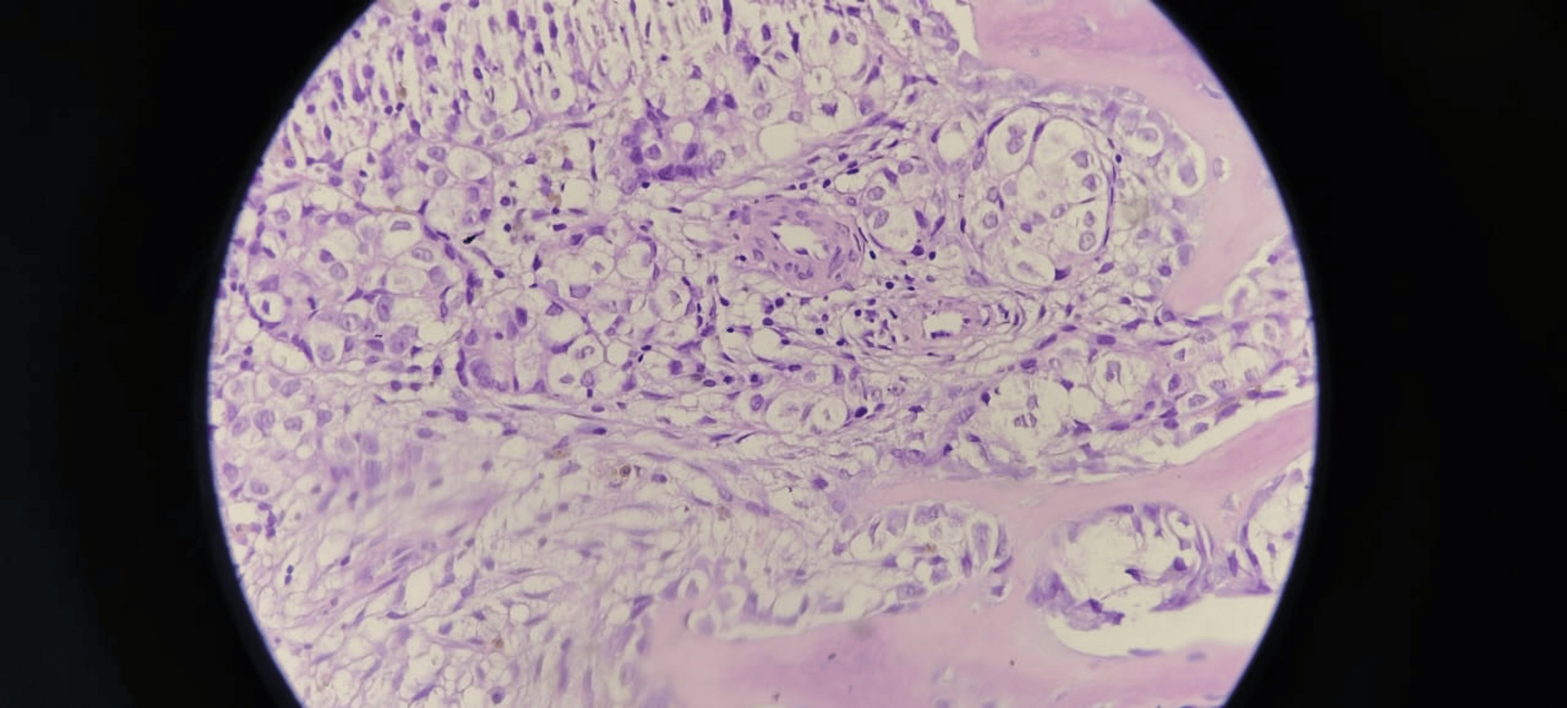
Bone marrow biopsy showing the presence of metastasis (hematoxylin and eosin, ×400).

Bone marrow examinations were performed in relation to the following reasons: unexplained anemia in 185 patients, suspected multiple myeloma in 87 patients, pyrexia of unknown origin in 43 patients, unexplained leukocytosis in 27 patients, and unexplained pancytopenia in 25 patients. Bone marrow biopsies were also checked against a multitude of indicators, which included assessment of age-adjusted norms for cell and cellularity as well as quantitative and qualitative assessments of lineage-specific erythroid, myeloid, and megakaryotic series. Special emphasis was given to the evaluation of plasma cells, which yields quantification under mildly increased (≤10%), moderately increased (11-59%), or significantly increased (≥60%) categories (Figure 2). The classification of dysplastic features included erythroid and myeloid dysplasia, such as nuclear budding, internuclear bridging, karyorrhexis, hypogranularity, nuclear hyposegmentation, and megakaryocytic dysplasia, comprising micromegakaryocytes and hypolobulated forms. Other important orthopedic diagnoses included metastatic deposits, chronic myeloproliferative neoplasms, chronic lymphoproliferative disease, amyloidosis, and renal bone disease (Figure 3).

This method facilitated a comprehensive assessment of hematological changes in CKD, allowing the differentiation between anemia of CKD and other causes of anemia. It also enabled the identification of statistical relationships between the stages of CKD and certain bone marrow findings and provided important details about the presentation of CKD in the local population. It enabled clinical guidance for clinical decision support to be developed and contributed to the optimization of understanding for treatment beyond conventional methodologies. In-depth evaluation showed that 55.9% of patients with CKD had some abnormality of the bone marrow, which underscores the relationship between renal impairment and pathological changes in the bone marrow.

Statistical analysis

Frequencies and proportions were summarized. Hypothesis testing used chi-square goodness‑of‑fit, chi-square test for independence (when cross‑tabulating CKD stage vs. marrow findings), and two‑tailed binomial confidence intervals (Clopper‑Pearson). Analyses were performed using Python 3.11 and Microsoft Excel. P-values <0.05 were considered statistically significant.

Ethical considerations

Patient confidentiality was maintained throughout data collection and analysis. All personal identifiers were removed from the dataset before analysis. The study was conducted following the ethical principles of the Declaration of Helsinki and Good Clinical Practice guidelines.

## Results

Demographic and clinical characteristics

Of the 220 CKD patients enrolled in the study, 139 (63.2%) were male, and 81 (36.8%) were female (Table 1). Most patients (138, 62.8%) were in the age range of 41-60 years, and the most populous age group was 51-60 years (85, 38.7%) (Table 2). Comorbidities were highly prevalent. Hypertension was seen in 198 (90%) patients, and diabetes mellitus was noted in 102 (46.4%) patients. The most commonly reported clinical symptom was weakness/fatigue (166, 75.5%) (Table 3).

**Table 1:** Gender distribution of patients.

Gender	Number (n = 220)	Percentage
Male	139	63.2
Female	81	36.8

**Table 2:** Distribution of patients across various age groups.

Age (in years)	Number (n = 220)	Percentage
10–20	2	0.9
21–30	8	3.6
31–40	30	13.6
41–50	53	24.1
51–60	85	38.7
61–70	34	15.5
71–80	8	3.6

**Table 3:** Frequency of various clinical features and symptoms reported in chronic kidney disease patients.

Feature/Condition	Number
Fever	27
Weight loss	25
Weakness/Lethargy/Fatigue	166
Back pain	52
Diarrhea	5
Vomiting	3
Headache	9
Burning micturition	6
Oliguria	15
Hepatomegaly	15
Splenomegaly	12
Lymphadenopathy	10
Petechiae/Purpura	8
Alcoholism	14
Diabetes	102
Hypertension	198
Ischemic heart disease	3
Hypothyroidism	2
K/C/O malignancy	8

Hematological parameters

All patients had anemia, with the moderate subtype being the most common, seen in 101 (45.9%) patients, followed by severe anemia in 84 (38.2%) patients, and mild anemia in 35 (15.9%) patients (Table 4). The dominant morphological type was normocytic normochromic anemia, seen in 107, (48.6%) patients, followed by dimorphic anemia in 51 (23.2%) patients, microcytic hypochromic anemia in 40 (18.2%) patients, and macrocytic anemia in 22 (10.0%) patients (Table 5).

**Table 4:** Severity of anemia. WHO cut‑offs, g/dL: mild ≥10–11.9, female; ≥10–12.9 male; moderate: 8–9.9; severe <8.

Grade of Anemia	Number	Percentage
Mild	35	15.9
Moderate	101	45.9
Severe	84	38.2

**Table 5:** Anemia types observed in chronic kidney disease patients based on red cell morphology.

Type of anemia	Number	Percentage
Normocytic normochromic anemia	107	48.6
Microcytic hypochromic anemia	40	18.2
Macrocytic anemia	22	10
Dimorphic anemia	51	23.2

Based on total leukocyte counts (normal range = 4,000-11,000 × 10³/µL), Most patients (160, 72.7%) had normal total leukocyte counts (4,000-11,000 × 10³/µL), while 33 (15.0%) had leukopenia, and 27 (12.3%) had leukocytosis (Table 6). Thrombocytopenia (<150 × 10³/µL) was present in 47 (21.4%) patients (Table 7).

**Table 6:** Distribution of total leukocyte counts in the study population.

Total leukocyte count (×10³/µL)	Number	Percentage
<4,000	33	15
4,000–11,000	160	72.7
>11,000	27	12.3

**Table 7:** Platelet count (×10³/µL) range observed in chronic kidney disease patients undergoing bone marrow examination.

Platelet count range	Number	Percentage
<20,000	3	1.4
20,000–50,000	11	5
50,000–1 lakh	14	6.4
1 lakh–1.5 lakh	19	8.6
1.5–5 lakh	173	78.6

Bone marrow findings

The indications for bone marrow examination in CKD patients are shown in Table 8. In total, 185 (84.1%) patients had unexplained anemia, 87 (39.5%) patients had probable multiple myeloma, and 43 (19.5%) patients had pyrexia of undetermined etiology. Additional indications included unexplained cytopenias and suspicion of malignancy.

**Table 8:** Clinical indications for performing bone marrow examination in chronic kidney disease patients.

Indication	Number
Unexplained anemia	185
Unexplained thrombocytopenia	22
Unexplained thrombocytosis	2
Unexplained leucopenia	8
Unexplained leukocytosis	27
Unexplained pancytopenia	25
Pyrexia of unknown origin	43
Suspected multiple myeloma	87
Suspected metastasis	8

**Figure 4:**
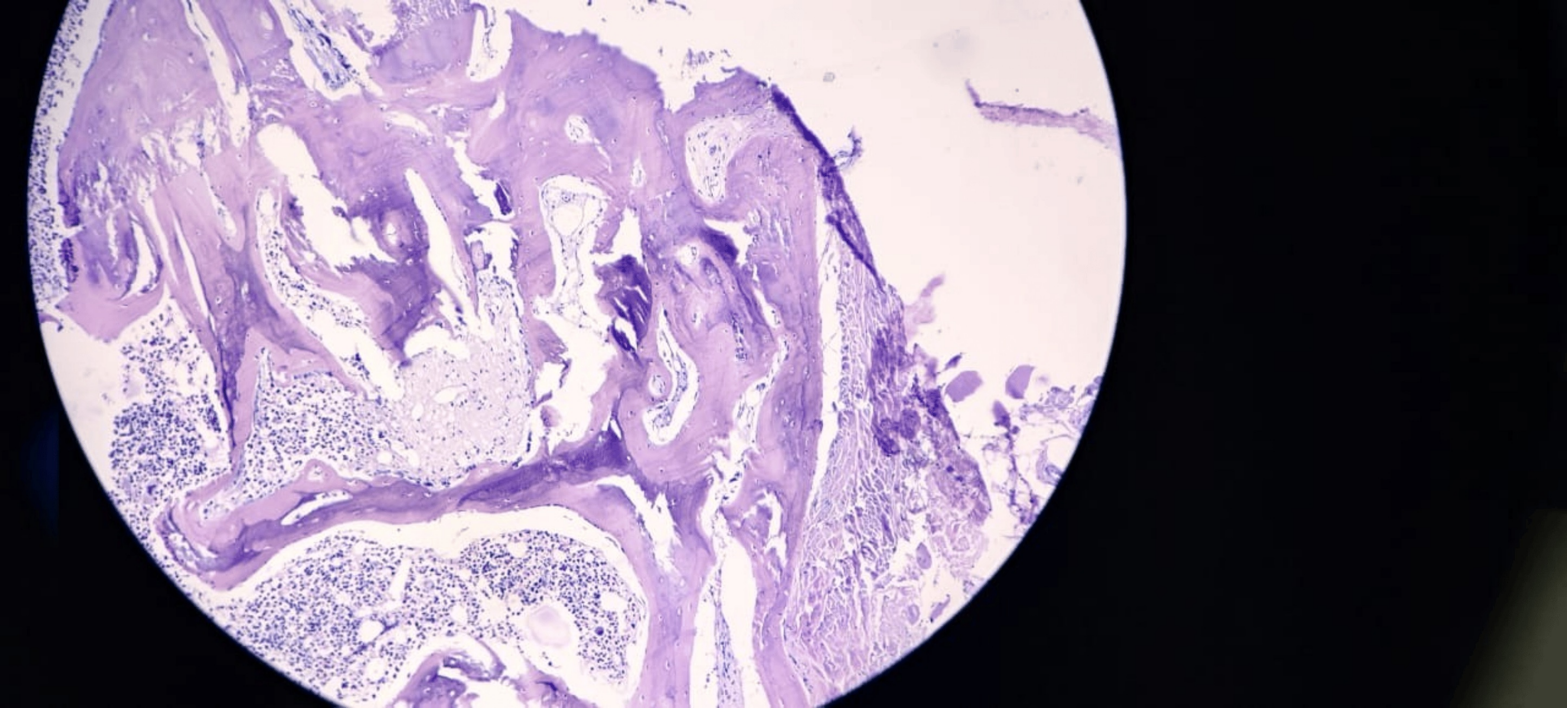
Bone marrow biopsy in renal osteodystrophy showing thickened trabeculae (hematoxylin and eosin, 100×).

**Figure 5:**
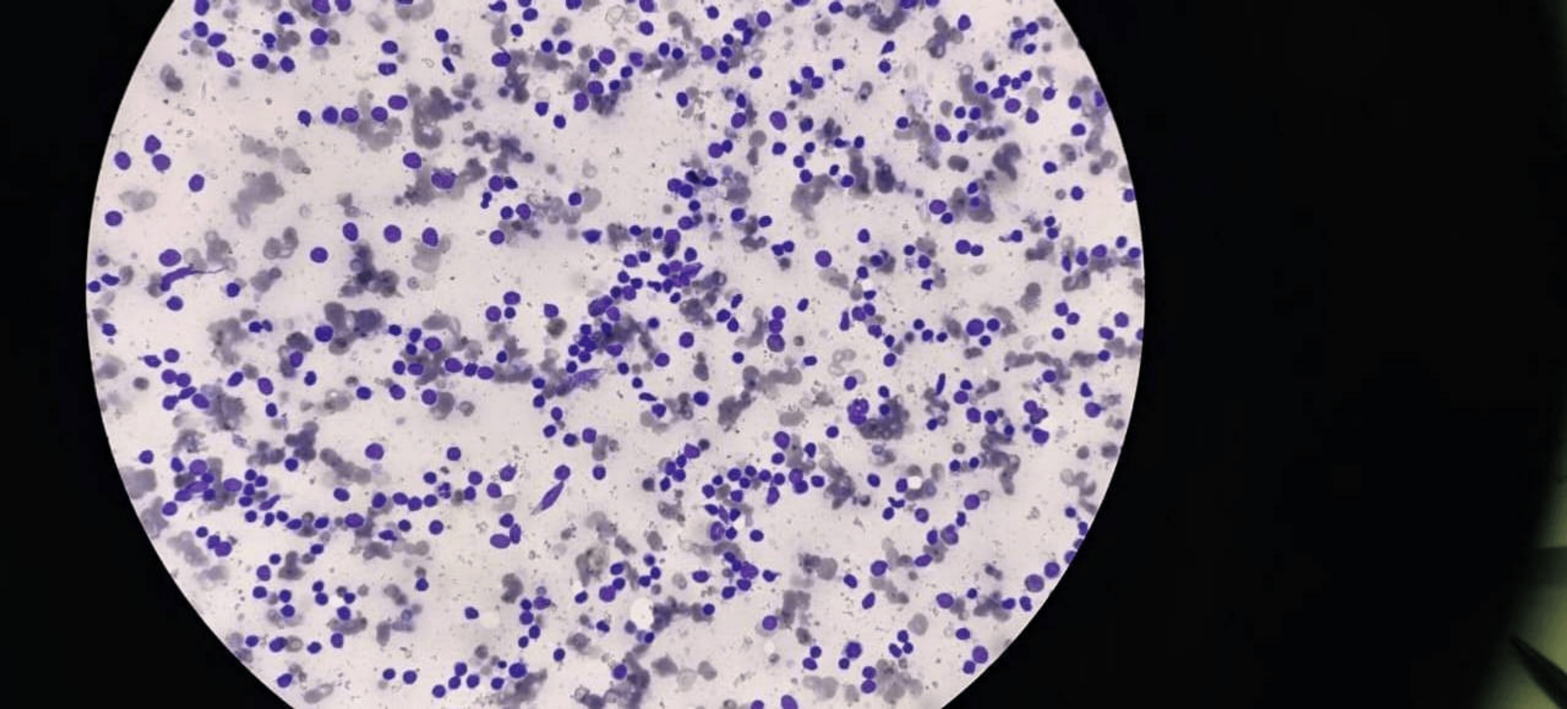
Bone marrow aspirate showing the presence of mature lymphoid cells in a case of chronic lymphoproliferative disease (Leishman, ×400).

Ordinary trifunctional hematopoiesis was the most commonly noted marrow specimen abnormality in 97 (44.1%) patients (Table 9). Increased plasma cells were one of the more prominent pathological findings seen in 51 (23.2%) patients. These were further categorized to ≤10% in 28 patients, 11-59% in 17 patients, ≥60% in six patients. Features of dyspoiesis were seen in 18 (8.2%) patients, while erythroid hyperplasia features were observed in 39 (17.7%) patients. Other less frequent findings included myeloid hyperplasia (3.3%), chronic lymphoproliferative neoplasm (0.4%), renal bone disease (0.9%), and amyloidosis (0.4%) or chronic myeloproliferative neoplasm (0.4%) (Figures 4, 5).

**Table 9:** Bone marrow impression or diagnosis findings among chronic kidney disease patients.

Bone marrow impression/Diagnosis	Number	Percentage
Trilineage hematopoiesis	97	44.1
Increased plasma cells (≤10%)	28	23.2
Increased plasma cells (11–59%)	17	
Increased plasma cells (≥60%)	6	
Erythroid hyperplasia	39	17.7
Features of dyspoiesis	18	8.2
Myeloid hyperplasia	7	3.3
Metastasis	3	1.4
Chronic myeloproliferative neoplasm	1	0.4
Chronic lymphoproliferative disease	1	0.4
Amyloidosis	1	0.4
Renal bone disease	2	0.9

Association analysis

**Figure 6:**
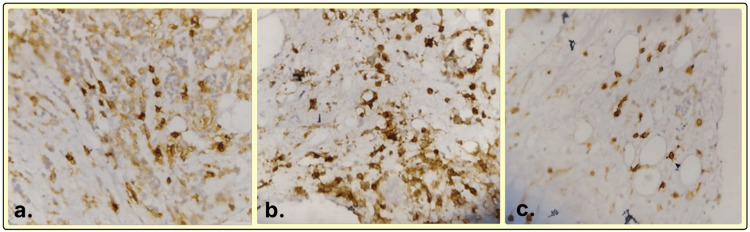
(a) Plasma cells showing CD38 positivity (immunohistochemistry, ×400). (b) Plasma cells showing CD138 positivity (immunohistochemistry, ×400). (c) Plasma cells showing kappa positivity (immunohistochemistry, ×400).

**Figure 7:**
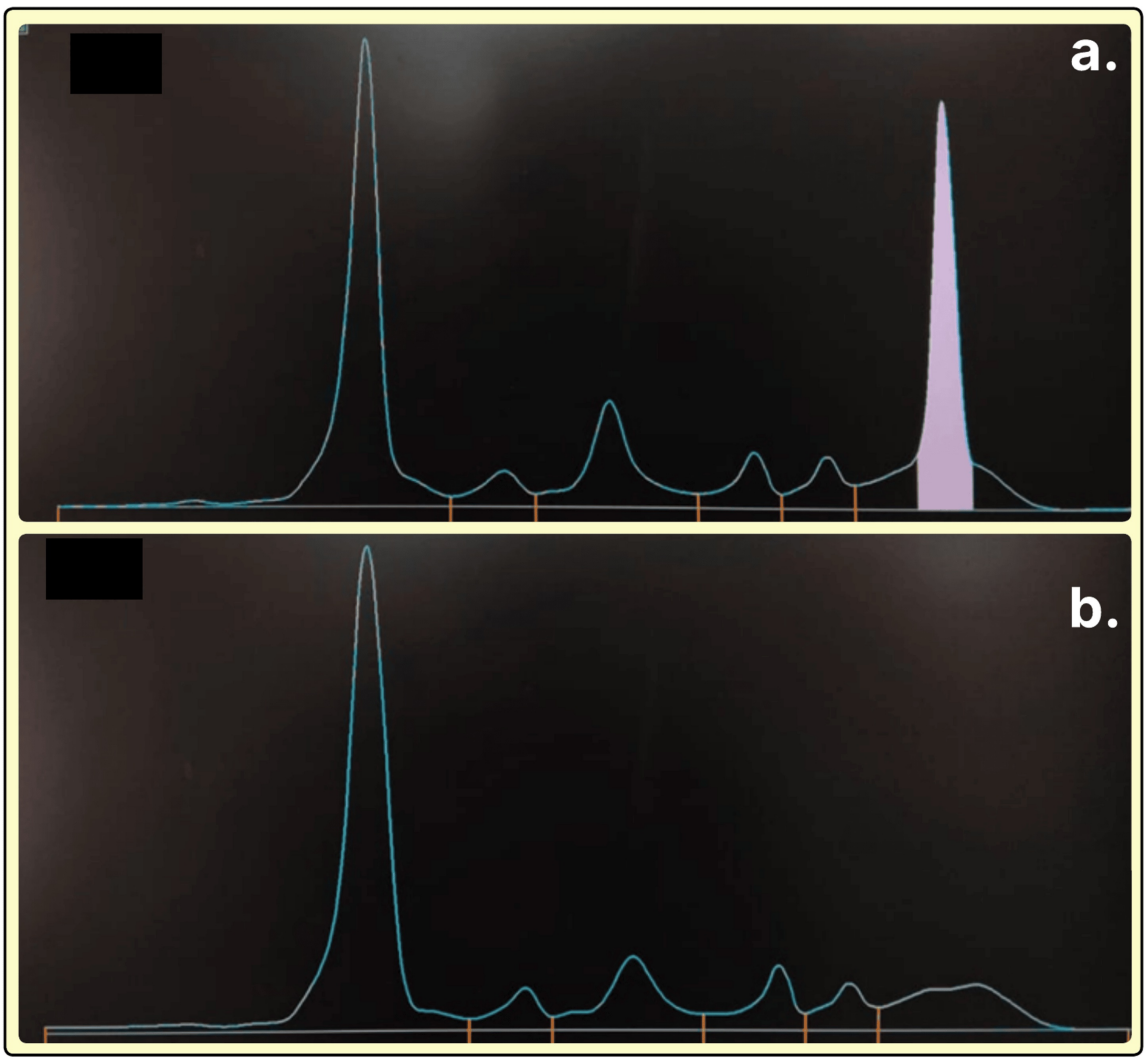
Serum protein electrophoresis densitograms. X axis: electrophoretic migration distance, progressing from the albumin fraction on the left through α1, α2, β, and, finally, the γ region on the right. Y axis: relative optical density (arbitrary units), proportional to protein concentration. (a) Monoclonal (M‑spike) peak in the γ region. (b) Broad distortion of the γ region.

Based on the study data, chi-square analyses revealed several significant associations in CKD patients with anemia. Different anemia types correlated with distinct bone marrow patterns: normocytic normochromic anemia frequently showed normal trilineage hematopoiesis, macrocytic anemia was associated with increased plasma cells, and microcytic hypochromic anemia was linked to erythroid hyperplasia. Anemia severity is significantly related to bone marrow cellularity, with severe anemia showing either hypercellular (compensatory) or hypocellular (marrow failure) patterns. The indication for bone marrow examination strongly predicted diagnosis: unexplained anemia cases often revealed normal hematopoiesis or erythroid hyperplasia, while suspected multiple myeloma cases showed significantly higher plasma cell percentages (Figure 6). Back pain in CKD patients tripled the likelihood of plasma cell disorders. Additional correlations emerged with CKD progression, showing declining normal hematopoiesis and increasing plasma cell disorders, erythroid hyperplasia, and dyspoietic features in advanced stages. Severe anemia cases demonstrated higher rates of plasma cell disorders and dyspoietic features compared to mild anemia. M-spike on serum protein electrophoresis strongly indicated increased bone marrow plasma cells (Table 10, Figure 7). Dialysis-dependent and older patients showed higher rates of plasma cell disorders and dyspoietic features with lower normal hematopoiesis compared to non-dialysis and younger patients. The detailed outputs of all statistical tests are summarized in the table (Table 11). All comparisons met the prespecified significance threshold (p < 0.05).

**Table 10:** Distribution of serum protein electrophoresis findings of patients.

Serum protein electrophoresis	Number
Normal	49
Distortion	42
M-spike	21

**Table 11:** Outputs of chi-square goodness-of-fit, chi-square test for independence, and exact binomial analyses showing the distribution of anemia grades, anemia morphology, leukocyte count categories, and the prevalence of selected bone marrow abnormalities among chronic kidney disease patients (n = 220). All tests met the prespecified significance threshold (p < 0.05).

	Test and rationale	Test statistic (df)	P-value	Interpretation
Anemia grades (mild/moderate/severe)	Chi-square goodness‑of‑fit vs. equal 33.3% expectation	32.0 (2)	1.1 × 10^‑7^	Distribution is not uniform; moderate anemia predominates
Anemia morphology (normocytic, microcytic, macrocytic, dimorphic)	Chi-square goodness‑of‑fit vs. equal 25% expectation	73.3 (3)	8.2 × 10^‑16^	Distribution is not uniform; normocytic‑normochromic predominates
Leukocyte count categories (<4,000/4,000–11,000/>11,000 µL)	Chi-square goodness‑of‑fit vs. equal 33.3% expectation	153.9 (2)	3.8 × 10^‑34^	Distribution is not uniform; normal range is the most common
Prevalence of selected marrow abnormalities	Exact 95% binomial CIs	—	—	Plasma cells, 23.2% (17.6–28.8); erythroid hyperplasia, 17.7% (12.7–22.8); dyspoiesis, 8.2% (4.6–11.8)

## Discussion

This comprehensive analysis of bone marrow findings in CKD patients from a tertiary care center in Bihar provides valuable insights into the hematological complications of kidney disease in this regional context. Several significant abnormalities in bone marrow were found in individuals with CKD. One of the most remarkable results was that over one-fourth (23.2%) of the group had elevated plasma cells, which may indicate an underlying plasma cell abnormality. This can be due to several reasons. First, the immunological perception linked with uremia may lead to increased plasma cell expansion [7]. Second, the shared etiological factors between CKD and plasma cell malignancies [8], especially among the older population, tend to explain this correlation. Third, renal impairment is a frequent problem accompanying multiple myeloma [9]. Therefore, these factors might explain the selection bias within the study population.

The exacerbated prevalence of plasma cells with the advancement of CKD stages may also be an indication of the influence of the uremic environment on the excessive proliferation of these cells. Recent literature suggests that chronic inflammation that accompanies kidney disease may provide a favorable niche for the development of plasma cell disorders [10]. Additionally, the strikingly elevated prevalence of plasma cell disorders in the cohort (23.2%) also surpasses the benchmarks from Western literature (approximately 10-15%), suggesting some regional or ethnic differences that should be studied further [11].

The presence of dyspoietic features in 8.2% of patients, increasing to 11.4% in Stage 5 CKD, suggests that uremic toxins may have a direct impact on hematopoietic stem cells and erythroid precursors. This hypothesis was further supported by the higher prevalence of dyspoiesis in dialysis-dependent patients (14.3%) compared to non-dialysis patients (6.7%) [12].

The findings support more recent studies, which showed that uremic toxins such as indoxyl sulfate and p-cresyl sulfate have the potential to cause oxidative stress and apoptosis to hematopoietic progenitor cells. This dyserythropoiesis would likely foster an increased requirement of erythropoietin, which is a frequent difficulty encountered while treating CKD in its advanced stages [13]. The fact that diabetic CKD patients presented with dyspoietic features at a higher rate (9.8%) than non-diabetic CKD patients (6.8%) illustrates that certain metabolic conditions might worsen the impact of uremia on bone marrow activity and function.

Erythroid hyperplasia, seen in 17.7% of patients, is a compensatory response to anemia in CKD. Enhanced erythroid hyperplasia in microcytic hypochromic anemia (25.0%) compared to other types of anemia suggests that iron deficiency drives increased erythropoiesis. On the other hand, the persistence of anemia regardless of the presence of erythroid hyperplasia suggests ineffective erythropoiesis, which may be due to uremic inhibitors or relative erythropoietin deficiency [13].

The stepwise increase in erythroid hyperplasia with advancing CKD stages (14.3% in Stage 3, 18.4% in Stage 4, 20.0% in Stage 5) illustrates the interchanging complex nature of these compensatory mechanisms. This pattern highlights the numerous factors impacting kidney function, erythropoietin synthesis, and bone marrow activity in the context of anemia associated with CKD.

These findings have several important clinical implications. First, the marked elevation of plasma cell disorder prevalence among CKD patients poses the risk multiplier for multiple myeloma in the subset with M-spike on serum protein electrophoresis; hence, testing for myeloma should be done aggressively in these patients [14]. Second, the correlation of bone marrow pathologies with the level of anemia suggests that more frequent routine blood tests for complete blood counts should be done at lower hemoglobin levels in patients with CKD, which is indicative of more advanced bone marrow involvement than would be expected for renal anemia. Third, cases of EPO resistance may be attributable to the presence of dysplastic changes, specifically in advanced CKD and dialysis patients. These patients may benefit from a more focused approach after bone marrow assessment [15]. Finally, the detection of metastatic cancer and hematological cancers in 2.2% of CKD patients illustrates the importance of bone marrow testing and the additional comorbidities that are overlooked in CKD patients [2].

The prevalence of CKD in India (17.2%) exceeds global averages, with significant regional and rural-urban variations. This study from Bihar provides valuable insights into the hematological complications of CKD in this underrepresented region. The high prevalence of moderate-to-severe anemia (84.1%) in our cohort exceeds national estimates (approximately 60-70%), potentially reflecting regional disparities in healthcare access, nutritional status, and environmental exposures.

The predominance of normocytic normochromic anemia (48.6%) is consistent with the typical pattern of renal anemia. However, the substantial prevalence of dimorphic anemia (23.2%) and microcytic hypochromic anemia (18.2%) highlights the multifactorial nature of anemia in this population, likely involving iron deficiency, folate deficiency, and chronic inflammation, beyond the erythropoietin deficiency traditionally associated with CKD.

This study has several important limitations that warrant consideration. First, as a single-center retrospective study, selection bias represents a significant concern. Patients referred for bone marrow examination likely represent a subset with more severe or atypical presentations, potentially overestimating the prevalence of certain findings such as plasma cell disorders or dyspoietic features in the general CKD population. Second, the lack of a control group limits our ability to differentiate CKD-specific bone marrow changes from age-related or comorbidity-related changes, particularly because our cohort had a high prevalence of diabetes (46.4%) and hypertension (90%). Third, while we observed associations between uremia and various bone marrow abnormalities, we cannot establish direct causality without mechanistic studies to elucidate the pathophysiological pathways involved. Our suggestions regarding uremic toxins affecting hematopoietic stem cells remain hypothetical and require validation through prospective studies with uremic toxin quantification and in vitro experiments. Fourth, the cross-sectional nature of our study precludes analysis of temporal changes in bone marrow findings with disease progression or therapeutic interventions. Finally, limited cytogenetic and molecular studies may have underestimated the prevalence of clonal hematopoietic disorders in this population. These limitations highlight the need for multicenter prospective studies with appropriate controls and mechanistic components to further validate our findings.

## Conclusions

This in-depth study of bone marrow changes in CKD patients from Bihar uncovers a myriad of hematological findings that go well beyond the domain of renal anemia. The significant association between increasing stages of CKD and bone marrow changes suggests a relationship between disease progression and hematopoietic alterations. However, the cross-sectional design of this study precludes establishing causal relationships. The remarkable relative frequency of plasma cellular disorders and some degree of defective red blood cell production, especially in the more advanced stages of CKD and among patients undergoing dialysis, suggests that bone marrow examination may be clinically valuable in selected cases of advanced CKD with unexplained cytopenias or suspected paraproteinemias. Longitudinal studies would be needed to establish whether these bone marrow alterations are direct consequences of uremia or represent comorbid conditions that frequently accompany CKD. This study provides a useful baseline reference for future research and clinical practice by highlighting the dearth of previous data on CKD patients in Bihar and emphasizing the necessity for increased integration of hematological assessment in CKD care within this region.
